# Treatment of Cachexia in Gastric Cancer: Exploring the Use of Anti-Inflammatory Natural Products and Their Derivatives

**DOI:** 10.3390/nu16081246

**Published:** 2024-04-22

**Authors:** Jerocin Vishani Loyala, Billy Down, Enoch Wong, Benjamin Tan

**Affiliations:** 1 Queen Elizabeth Hospital, University Hospitals Birmingham NHS Foundation Trust, Birmingham B15 2GW, UK; 2 High Wycombe Hospital, Buckinghamshire Healthcare NHS Trust, High Wycombe HP11 2TT, UK; billy.down@nhs.net; 3 Institute of Immunology and Immunotherapy, University of Birmingham, Birmingham B15 2TT, UK; b.tan.1@bham.ac.uk

**Keywords:** gastric cancer, cachexia, natural products, anti-inflammatory

## Abstract

(1) Background: Gastric cancer is a significant cause of cancer-related mortality worldwide. Weight loss and malnutrition associated with cancer are linked with increased mortality rates and reduced quality of life. Cancer cachexia, characterised by the loss of skeletal muscle, is associated with approximately 20% of cancer-related deaths and differs from malnutrition in that it cannot be fully reversed by nutritional support alone. It is now recognised that the primary pathophysiological process underlying cancer cachexia is chronic inflammation leading to increased calorie consumption. Current treatments that focus on nutritional supplementation, psychological counselling, appetite stimulation and reducing inflammation are lacking in efficacy. This review focuses on the evidence supporting the potential roles of natural anti-inflammatory products and their derivatives including fatty acids, probiotics, amino acids, curcumin, fucoidan, epigallocatechin-3-gallate, ginger, resveratrol and Boswellia serrata in the management of gastric cancer cachexia. (2) Results: While natural anti-inflammatory products show promise in a number of in vitro and in vivo studies, there are only a small number of human studies available. Where present, the evidence base is heterogeneous, with varying study methodologies and outcomes. (3) Conclusions: Natural anti-inflammatory products represent a potential adjunctive therapy for gastric cancer cachexia. Further research, particularly well-designed clinical trials, is needed to elucidate their optimal role, dosing and safety profiles in the management of gastric cancer cachexia.

## 1. Introduction

Gastric cancer (GC) is one of the leading causes of cancer-related death with an incidence highly variable by geographic location [1]. The highest incidence rates are seen in eastern Asia and eastern Europe and the lowest rates in northern Europe and northern America. Risk factors include Helicobacter pylori infection, family history and lifestyle factors including tobacco and alcohol consumption [2]. Treatment modalities vary depending on the extent of the disease. Surgical resection with or without peri-operative chemotherapy with curative intent for localised disease has a 5-year survival of 70% [1,3]. In advanced disease, treatment is with palliative chemotherapy and 5-year survival is 7% [4].

Weight loss and malnutrition are commonly associated with cancer with up to 80% of patients becoming malnourished during their disease progress [5]. The most common cause of weight loss is malnutrition, which is an imbalance between the individual’s requirements and their nutritional intake. It can lead to a number of conditions including low body mass, marasmus and kwashiorkor. These conditions differ from cachexia in that they can be reversed with adequate nutritional intake. The pathophysiology of cachexia also differs from malnutrition, with the primary driving factor being inflammation due to underlying disease.

Cachexia is defined as a complex metabolic response with loss of skeletal muscle with or without loss of adipose tissue [6,7,8]. It is associated with a number of non-malignant chronic diseases including heart failure, chronic obstructive pulmonary disease and auto-immune deficiency syndrome (AIDS) as well as malignancy [6]. Cancer cachexia, defined by international consensus, is a multifactorial syndrome with continuous skeletal muscle loss with or without loss of fat that conventional nutrition cannot fully reverse and has been demonstrated to be responsible for more than 20% of cancer-related deaths [5,9]. This loss of skeletal muscle to generate calories is an adaptive response to the increased metabolic demand of systemic inflammation—an attempt by the body to redirect energy to vital organs including the brain [10].

**Figure 1:**
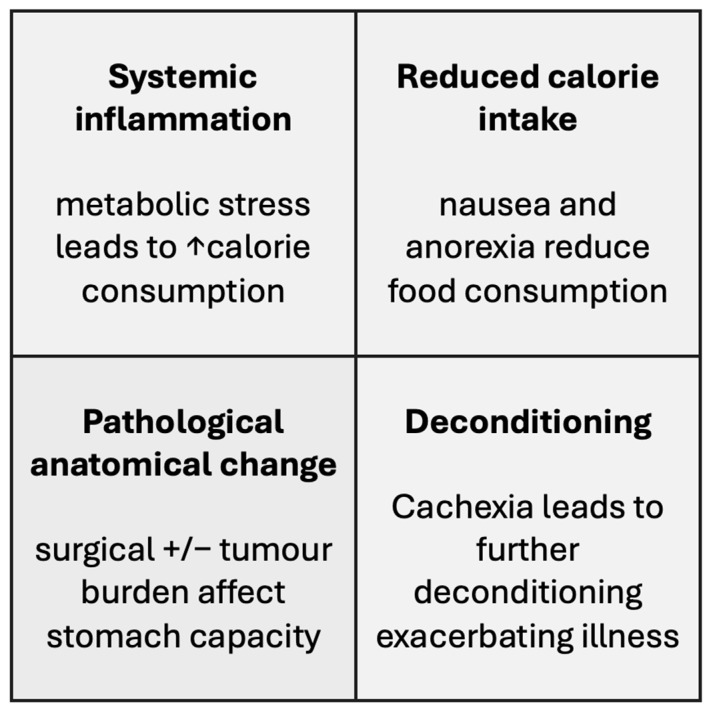
Pathological effects of gastric cancer cachexia.

Figure 1 summarises the pathological effects of gastric cancer cachexia. Cancer cachexia was traditionally thought to be due to malnutrition but is now known to be largely due to the metabolic stress of systemic inflammation, which increases calorie consumption to a level not reversible with increased nutritional intake and results in loss of lean body mass [6,7,8]. This is further compounded in many patients by reduced calorie intake due to symptoms such as nausea and anorexia, which may be associated with the underlying disease process and/or with adverse effects of treatments including chemotherapy [11]. This combination of abnormally high metabolism combined with reduced food intake results in negative nitrogen and energy balance, which leads to the catabolism of lean tissue and adipose tissue to preserve energy supply to the vital organs, driving cancer cachexia [8]. Patients with gastric cancer are at a further increased risk of malnutrition and cachexia as the cancer involves the stomach—the primary digestive organ. This can further lead to reduced intake and nutrient malabsorption secondary to the disease process or treatments such as surgical resection or radiotherapy [5]. The result of these pathological effects is loss of body mass and deconditioning, which leads to increased mortality rates and reduced quality of life [11,12].

The pathophysiology of cachexia is due to chronic systemic inflammation powered by pro-inflammatory cytokines such as tumour necrosis factor-alpha (TNF-a) and interleukin 6 and 1 (IL6, IL1) from tumour and host cells. Mainly these cytokines affect the muscle tissues but they also cause multi-organ dysfunction affecting adipose tissue, the liver, the gastrointestinal tract, blood, the brain and the heart [11,12].

Cancer cachexia has been demonstrated to have a negative impact on patient quality of life outcomes and is associated with increased chemotherapy toxicity and adverse effects, as well as increased complications following surgery [8,11,13]. These effects lead to a shorter life expectancy, with cancer cachexia associated with increased mortality risk [14].

Cancer cachexia is a complex disease process with no set standard for clinical assessment [6]. There are a number of clinical tools available to screen for patients at risk such as the Nutrition Risk Screening 2002, Malnutrition Universal Screening Tool and Patient-Generated Subjective Global Assessment tool adapted for patients with cancer, but there are concerns regarding the accuracy of estimating real skeletal muscle loss [6,14,15]. There is a potential role for the use of biomarkers in the diagnosis of cancer cachexia—human CC patients have been demonstrated to have elevated C-reactive protein (CRP), fibrinogen, IL1, IL6 and TNF-a [6,8]. A high score in the modified Glasgow prognostic score (mGPS) is associated with poor prognosis in advanced cancer and utilises increased serum fibrinogen and CRP and decreased serum albumin levels, demonstrating the centrality of systemic inflammation [6,8]. The gold standard for body composition assessment is computed tomography or magnetic resonance imaging of the 3rd lumbar vertebra cross-section; however, these investigations are not cost-effective, and computed tomography imposes ionising radiation risk [6,8].

**Figure 2:**
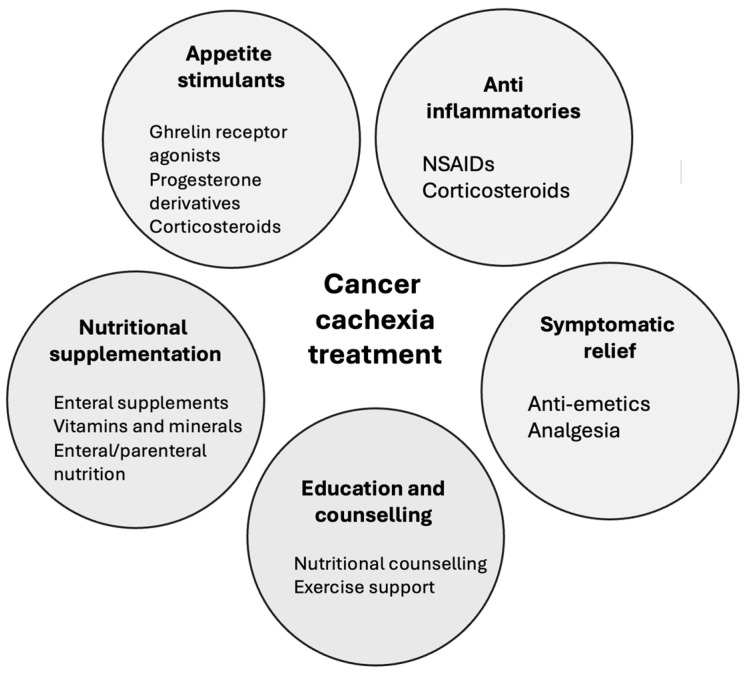
Current cancer cachexia treatment modalities.

The aim of cancer cachexia treatment is the prevention of muscle loss through a reversal of the pathological negative energy balance [6]. The mainstay of treatment is increasing nutritional intake through nutritional supplements and dietary counselling [16]. Pharmacological treatment is not universal but includes medications aimed at stimulating appetite and/or reducing inflammation [6] (Figure 2).

Initial approaches to management involve oral nutritional supplements with some cases requiring enteral or total parenteral nutrition [6,17]. Pharmacological treatments include short courses of glucocorticoids (reducing inflammation and stimulating appetite) and progesterone derivatives; however, the former comes with a catabolic effect on skeletal muscle and the latter with an increased venous thromboembolism risk [6,13] (Figure 2). They have the potential to inhibit pro-inflammatory cytokines that are part of cancer cachexia such as TNF-a and IL1. Non-steroidal anti-inflammatories (NSAIDs) may be used alongside steroids but are not tolerated by those with asthma and known gastrointestinal problems. The risk of toxicity limits its use, as chronic usage can lead to gastric ulcerations, perforation and even obstruction [18]. NSAIDs also have the potential to reduce TNF-a levels and increase lean weight; however, they have not been regularly used outside of clinical trials. Another downside of pharmacological treatment is that they can interact with chemotherapy agents negatively and cause adverse effects on patients, and in some cases, pharmacological treatments add further medication burden on patients [14].

The recognition of the importance of reducing systemic inflammation in the treatment of cancer cachexia underpins the rationale for the recent interest in the potential role of natural anti-inflammatory products. Natural products given in calculated doses are considered nutritional interventions or natural dietary supplements, defined as products taken orally that contain vitamins, minerals, herbs or any other substance that can supplement the diet [15,16]. The supplements are not intended to treat, diagnose, cure or alleviate the effects of the disease as per the regulations of the Food and Drug Administration and so are not regarded as pharmacological therapy [19]. Therefore, the benefits are that they are often cost-effective, readily available and generally well-tolerated [20].

Gastric cancer is a leading cause of cancer-related death with a 5-year survival of 7% in advanced cases. Cancer cachexia, the pathological loss of skeletal muscle with or without loss of fat that cannot be fully reversed by conventional nutrition, is thought to be responsible for 20% of cancer-related deaths. Systemic inflammation is now known to be the central underlying causative factor, and treatment options are limited, with the mainstays including nutritional supplementation and nutritional counselling, as well as the use of appetite stimulants and anti-inflammatories though their use varies by institution. Natural dietary supplements may provide adjunctive therapies for patients with gastric cancer cachexia. This review will focus on the evidence in the literature and the potential roles of the more widely studied natural anti-inflammatory products in the treatment of gastric cancer cachexia.

## 2. Natural Anti-Inflammatories and Their Derivatives

### 2.1. Introduction

Natural anti-inflammatories and their derivatives are recognised as having a potential role in the mitigation of cancer cachexia, which is now understood to be primarily driven by systemic inflammation causing pathological levels of muscle wasting. Table 1 provides a summary of the natural anti-inflammatory compounds with their major dietary sources and proposed mechanisms of action.

Essential fatty acids are found abundantly in sources including fish, flaxseeds and walnuts and possess anti-inflammatory properties [21]. Probiotics are present in fermented foods such as kefir, kimchi and kombucha and may modulate the gut microbiota with a potential result in reducing inflammation [22,23]. Essential amino acids are present in proteinaceous foods such as meat, eggs and seeds and are crucial for muscle protein synthesis and may therefore help promote muscle anabolism and reduce catabolism [24,25]. Curcumin, found in the commonly used flavouring turmeric (*Curcuma longa*), and fucoidan, extracted from brown algae, demonstrate anti-inflammatory effects and may improve chemotherapy tolerance [26,27,28]. Epigallocatechin-3-gallate (EGCG) is present in green and white tea, gingerol in ginger, quercetin in fruits and vegetables, resveratrol in red grapes and capsaicin in chili peppers [29,30,31]. Their mechanisms are unknown; however, they may exhibit anti-inflammatory properties that may alleviate systemic inflammation in cancer cachexia reducing overall metabolic burden. We will now discuss these compounds in more detail.

### 2.2. Essential Fatty Acids

Fatty acids (FAs) are a component of fat and are found at a cellular level in membrane lipids [16,32]. Fatty acids can be divided into saturated and unsaturated depending on the presence of double bonds between hydrocarbons. Most fatty acids can be synthesised by the human body; however, polyunsaturated fatty acids (PUFAs) need to be obtained from external sources; these are also known as essential fatty acids. From PUFAs, humans can then further synthesise eicosapentaenoic acid (EPA) and docosahexaenoic acid (DHA), which are omega-3 FAs, but they can also be sourced from fish and marine products. EPA and DHA are mostly derived from fish oil [33].

EPA and DHA have been shown in some studies to improve cancer cachexia by inhibiting proinflammatory cytokine production [16,19]. Eicosapentaenoic acid also modulates the production of prostaglandins and leukotrienes. One mechanism is by reducing the availability of arachidonic acid, an omega-6 fatty acid, for cyclooxygenase and lipoxygenase enzymes through competitive inhibition [34]. This reduces the downstream products from arachidonic acid metabolism. Furthermore, the prostaglandins derived from EPA are less pro-inflammatory than those produced using arachidonic acid [34]. FAs have also been shown to reduce acute phase proteins such as C-reactive protein in different types of cancer [20,21].

In a gastrointestinal cancer study by Shirai et al., skeletal muscle mass and lean body mass significantly increased with fish oil-enriched nutrition given during systemic chemotherapy to patients [28]. It also showcased that in patients with modified Glasgow performance score (mGPS) of 1 or 2, fish oil-enriched nutrition was associated with improved tolerance to chemotherapy and improved prognosis when compared to a control group not supplemented with fish oil-enriched nutrients. In a small study in Sweden of 24 patients with advanced gastrointestinal cancer, Persson et al. demonstrated that fish oil has the potential to have weight stabilisation or weight gain properties compared to patients on melatonin alone, 38% of the fish oil group showed weight stabilisation [35].

The main limitations of these studies are that there are not many in the literature, and those that exist vary widely in dosage and patient cohort, particularly in terms of inflammatory-oxidative stress status prior to treatment [36]. They are also limited by short treatment and follow-up periods and low dosages of fatty acids. The recommended minimum dosage of EPA is 2 g/per day and longer than 2 months [32]. There is some early evidence to suggest a possible role for fatty acids in the treatment of cancer cachexia; however, further evidence is needed to support randomised controlled trials in gastric cancer cachexia before hypotheses can be confidently formulated [37].

### 2.3. Probiotics/Gut Bacteria

Systemic inflammation has been demonstrated to be central in the process of cancer cachexia, increasing the overall metabolic demand and outstripping calorie and nutrient intake. The intestinal microbiota comprises approximately 100 trillion microorganisms including bacteria, fungi and viruses [38]. The majority of the microbiota is within the gastrointestinal tract. Animal models have been used to demonstrate associations between the intestinal microbiota and systemic inflammation in a number of diseases including obesity, cardiovascular disease, inflammatory bowel disease and cancer, but the mechanisms underlying the link between the human gut microbiota and systemic inflammation in cancer cachexia patients is not yet fully understood [17].

Dysbiosis is a term that refers to harmful alterations of the microflora within the gastrointestinal tract and can be associated with changes in diet, use of antibiotics, disease states and other causes [17,38]. There are a number of proposed hypotheses linking dysbiosis with systemic inflammation including intestinal barrier dysfunction, increases in pro-inflammatory cytokine production, reduction in short-chain fatty acid production and production of pro-inflammatory metabolites [17,38,39].

The intestinal microbiota has been demonstrated to shift toward a dysbiotic state in animal models of cancer cachexia versus healthy controls [17]. This was linked to weight loss and muscle atrophy. Furthermore, in a study of human patients with cachexia secondary to advanced gastric cancer, significantly increased levels of intestinal barrier dysfunction were demonstrated, as well as significant differences in the gut microbial profile [40].

In a study of patients with colorectal cancer, the administration of post-operative probiotics demonstrated a significant reduction in microbiota dysbiosis with a reduction in chemotherapy-related adverse effects [41]. Though not directly demonstrated, improved tolerance to chemotherapy may reduce the risk of development of sarcopenia and cachexia. This was further demonstrated in another study of patients with colorectal cancer who received probiotics for six months and demonstrated reduced pro-inflammatory biomarkers [42].

Probiotics, defined as “live microorganisms that, when administered in adequate amounts, confer a health benefit on the host”, have more recently been recognised as a potential treatment for cancer cachexia [23]. Through prevention of dysbiosis, probiotics may preserve the integrity of the intestinal barrier, preventing translocation of bacteria and subsequent systemic inflammatory states, as well as maintaining higher levels of compounds such as short-chain fatty acids, which rely on intestinal bacteria activity for the formation [13]. There are no studies in gastric cancer animal models or human patients, but a number of studies of non-gastric cancer-related cachexia exist. In one study of *L. reuteri* administered orally to a well-validated colon cancer gene knockout mouse model, treatment was associated with increased muscle mass and reduced muscle atrophy, as well as reduced markers of inflammation [43]. This was also demonstrated in a mouse model of cachexia in leukaemia [44].

There clearly is a paucity of evidence available for the role of probiotics in cancer cachexia, and the relevance of these findings in mouse models on human patients is not yet known. Animal models typically demonstrate aggressive cancer phenotypes with rapidly progressing cachexia; so further investigation in human studies is needed, ideally in the form of a randomised controlled trial. The studies demonstrate a possible role of probiotics in reducing inflammation, which may reduce the overall metabolic burden of homeostasis, as well as reducing side effects of cancer treatment, which may improve appetite and increase calorie intake, which will reduce cachexia. There have been some concerns over the safety of probiotic administration in cancer patients, given their often immunosuppressed state and the dangers of administering live bacteria. A systematic review showed that there are rare isolated reports of possible sepsis following probiotic use in cancer patients, which may need further investigation [45]. This would need to be considered if planning a randomised controlled trial.

### 2.4. Amino Acids

Skeletal muscle loss is an integral part of cancer cachexia, leading to an interest in the potential role of amino acids in its treatment. Essential amino acids (EAAs) are amino acids that cannot be synthesised by the body and have to be sourced exogenously. Branched-chain amino acids (BCAAs) are a special group of EAAs that consist of valine (Val), leucine (Leu) and isoleucine (Ile), which are needed for skeletal muscle production and also serve as substrates for tumour growth. Malignant tumours require a constant source of glucose and glutamine due to high metabolic activity, which increases in size as the tumour grows. This deprives the muscles of their nutrients, which causes muscle fibres to alter their metabolism by catabolising muscle into amino acids to support tumour growth and liver metabolism [16,31]. It was also noted that cancer patients had markedly altered circulating amino acids, especially EAAs, and this varied depending on patients’ weight loss, nutrition and metastasis, which is why plasma amino acids could play a potential role as biomarkers for diagnosis and screening cancer patients [46]. Miyagi et al. demonstrated that gastric cancer had reduced plasma amino acids [34,47]. Over time, however, anabolic resistance is seen in cancer cachexia in advanced disease—this is where the body’s tissues become less responsive to anabolic stimuli such as external amino acids. This process contributes to muscle wasting and is one of the reasons that nutritional support alone cannot reverse cancer cachexia [33,47].

Leu supplementation has the potential to stimulate muscle anabolism and reduce catabolism, especially β-hydroxy-β-methylbutyrate (HMB), a metabolite of Leu [48]. Amino acids have been widely tested with animal models and have shown promising results in attenuating cancer cachexia. There have been no clinical trials in humans of single amino acids as a sole treatment for cancer cachexia, and they are usually tested along with anti-inflammatory and antioxidant mixtures [46]. Cangiano et al. studied 25 cancer patients, including 9 gastrointestinal cancer, comparing BCAAs (n = 13) with placebo (n = 12) and showed reduced cancer-associated anorexia. Patients’ nutritional status was compared pre- and post-intervention using biochemical testing and evaluation of daily caloric intake. Certain amino acids were noted to be higher in BCAA patients compared with placebo, and it was noted that caloric intake was increased in the BCAA group [49]. Yamamoto et al. administered 2.4 g of HMB to 22 patients with gastric cancer in addition to their daily protein and calorie intake for a duration of 16 days in a single-arm study. It also included a tailored exercise programme consisting of handgrip training, walking and resistance training. After 16 days, handgrip strength had increased with total calorie and protein intake compared to the previous study [35,37]. Although observations of single amino acids in animal models are encouraging, it is unknown whether this can be translated to humans, as animal muscle metabolism is different to humans and may cause a different reaction to amino acid supplementation [50].

In humans, Deutz et al. formulated a high leucine- and protein-rich diet, which was demonstrated to stimulate muscle protein synthesis in a group of 25 patients with mostly lung or colon cancer [46]. The authors hypothesised that the observation of a reduction in muscle breakdown was at least partly attributable to the increased intake of the essential amino acid leucine. It is notable however that the sample size is small, and a number of extra components were added to the treatment arm food including fish oil, protein, leucine and specific oligosaccharides. It is therefore difficult to determine the significance of the essential amino acid leucine specifically. Another study by Tayek et al. in a cohort of 10 patients with advanced intra-abdominally spread adenocarcinoma with a partial or complete obstructed gastrointestinal tract (of which 2 had gastric cancer) demonstrated that branched-chain amino acid-enriched total parenteral nutrition had a positive effect on protein metabolism. The cross-over study treated the subjects with a conventional total parenteral nutrition formula containing 19% branched-chain amino acid and an enriched formula containing 50% branched-chain amino acid in random order. Whole-body protein turnover was determined by continuous radiolabelled leucine infusion. Infusion of branched-chain amino acid-enriched total parenteral nutrition was associated with significantly increased whole-body protein synthesis (3.9 vs. 2.2 g protein per kg bodyweight per day; *p* < 0.005) versus the conventional total parenteral nutrition group. Nine out of ten patients showed improvement in albumin fractional synthetic rates while on a branched-chain amino acid-enriched feed, potentially improving overall nutritional status in this small group with advanced adenocarcinoma and inadequate oral intake [51].

The European Society for Clinical Nutrition and Metabolism provides guidelines for cancer cachexia management and advises a minimum of 1 g protein/kg/day based on an estimated 25 kcal/kg of daily energy expenditure [52]. This is now thought to be insufficient in patients with anabolic resistance and in those with significant muscle loss [48]. Further research is needed to understand the role of amino acids in treating gastric cancer cachexia.

### 2.5. Curcumin

Turmeric (*Curcuma longa*) is a part of the ginger family and contains the polyphenolic compound curcumin, which has long been used as a natural supplement. Curcumin has been reported to have anti-inflammatory, antioxidative and anticarcinogenic effects. Oelkurg et al. showed that curcumin was able to directly induce cell death in cancer cells in vitro [26]. The mechanism is thought to be via an increase in caspase activation.

Additionally, curcumin can stimulate appetite and may have direct effects on cancer cachexia. Like other natural remedies, the anti-cachectic property of curcumin was found to be related to its ability to suppress the activity of NF-κB by hindering phosphorylation and subsequent separation of one of its inhibitors, IκB, thereby potentiating its effects [26]. This pathway results in proteolysis in muscles but, in hepatocytes, results in the production of interleukin-6, 8 and C-reactive protein [53]. Aside from this pathway, curcumin also reduces the proteolytic effects of the 20S proteasome via suppression of proteolysis-inducing factor [41]. Elevated levels of IL-6 have also been implicated in cancer cachexia. Therefore, decreases in IL-6 activity via suppression of NF-κB by curcumin have shown benefits in counteracting the effects of cachexia in mice [26].

The anti-cachectic effects of curcumin have also been studied in a clinical trial. Chaiworramukkul et al. published a randomised, double-blind, placebo-controlled phase 2a study, where patients with solid malignancy were enrolled 1:1 to receive oral curcumin or placebo for 8 weeks [53]. No statistically significant changes were seen in body composition between the two groups. However, patients treated with curcumin had less reduction of hand grip strength although this was also not statistically significant. A small sample size probably limited the ability of this study to demonstrate a clear difference; so further research, both clinical and preclinical, is required to investigate the multiple effects of curcumin.

Curcumin is available as turmeric, but can also be obtained in concentrated forms as curcumoids (also containing demehtoxycurcumin and bis-demehtoxycurcumin) and curcumin alone. [54]. In clinical trials, curcumin is typically provided as an oral agent and can be provided in varying dosages. It has even been used as a supplement in conjunction with chemotherapy [55]. Systemic exposure of doses up to 8000 mg/d has been tolerated by patients in a clinical trial [56] Unfortunately, curcumin has poor oral absorption and weak bioavailability and is easily dissolved in aqueous solution. These properties may limit its effects when used as a supplement, so research is also required to determine the optimal delivery medium and method [57].

### 2.6. Fucoidan

Fucoidan products are carbohydrate compounds rich in fucose, which are found in brown, green and red marine macroalgae. Fucoidan compounds can be classified based on their molecular weight. Low- and middle-molecular-weight fucoidan products have been shown to increase cellular production of anti-oxidation and cytotoxicity while high-molecular-weight fucoidan is thought to enhance immune activity [27]. The multitude of activities is related to their structural diversity. The brown algae *Laminaria longipes* and *Saccahrina cichorioides* have been demonstrated to prevent the growth of cancer cells and sensitise these cells to X-ray radiation. Other fucoidan compounds found in other algae have been shown to induce apoptosis and augment mitochondrial membrane permeability in colon cancer cells [58].

More recently, work has been undertaken examining the immunomodulatory effects of fucoidan. It is thought that it does this by binding to and activating macrophages, monocytes and dendritic cells. In this way, an immune response is elicited and may assist in its antitumour activity [59].

Only one study looked specifically at advanced gastric cancer [60]. Twenty-four patients were administered high-molecular-weight fucoidan with chemotherapy, and these patients showed improved mean survival (12 vs. 8 months, *p* = 0.039) and longer chemotherapy treatment periods (7.4 vs. 4.6 months, *p* = 0.004) due to reduced fatigue levels. It is not quite understood how fucoidan is able to control fatigue caused by a chemotherapy agent. It is theorised that it could be due to fucoidan anticancer effects or their ability to suppress chemotoxicity.

Due to their varied structure and composition, research into their various effects is ongoing and complex. Although their many biological actions hold promise, clinical trials are currently limited [58].

### 2.7. Epigallocatechin-3-Gallate (EGCG)

Epigallocatechin-3-gallate (EGCG) is the major antioxidant found in green tea and possesses not only antioxidant properties but also anti-inflammatory, anti-mutagenic and anticarcinogenic properties. Wang et al. reported in a mouse model that EGCG was able to reduce the amount of body weight lost and the amount of muscle mass lost related to cancer, in a dose-dependent manner [29]. The pathway via which this occurred was shown to be by direct inhibition of nuclear factor—kappa light chain (NF-κB), with a subsequent downstream reduction in the ubiquitin–proteosome dependent proteolytic pathway, which normally results in enhanced protein breakdown. In the same study, EGCG decreased leucocyte infiltration and subsequently inflammation of skeletal muscle cells. These pathways may reflect a potential role for EGCG as an anti-cachexia agent.

It has also been recognised that the antioxidant capacity of other supplements such as vitamin C increased when combined with EGCG [60,61]. EGCG represents an important natural agent that requires further research in both pre-clinical and clinical studies. However, it has been highlighted that EGCG has low bioavailability, perhaps limiting its potential effects on humans [57].

### 2.8. Ginger

Ginger (*Zingiber officinale Roscoe*) has long been used in traditional remedies for a variety of ailments, particularly related to the gastrointestinal tract. It is hypothesised that the metabolites of ginger (gingerols and shogaols) act peripherally within the gastrointestinal tract to inhibit serotonin and cholinergic receptors, which subsequently improve gastric tone and motility [62]. Its use has been shown to improve gastric myoelectrical activity with improvements in symptoms of nausea, dysmotility and reflux-like symptoms in patients with advanced cancer. This subsequently may affect the anorexia–cachexia syndrome by improving the patient’s oral intake [30]. A wide variety of different cancers were included in the study, although none of the patients included had upper gastrointestinal cancers. However, all patients showed an improvement in symptoms suggesting this may be a generic beneficial effect, rather than for specific types of cancer. The same study reported no change in inflammatory markers for patients treated with 2 weeks of ginger [30].

### 2.9. Quercetin

Quercetin is a natural flavonoid found in fruits and vegetables such as onions, grapes, cherries, broccoli and citrus fruits [63]. It has antioxidant effects along with potential anti-cachexia properties. Oral supplementation with quercetin appears to have some beneficial effects against cancer cachexia. In an adenomatous polyposis coli mouse model study, mice given quercetin demonstrated less body weight reduction after 15 weeks compared with controls, along with less grip strength loss and less muscle mass loss [63]. Further clinical trials regarding the use of quercetin and its actions on cancer and cachexia are needed [64].

### 2.10. Resveratrol

Resveratrol is another naturally occurring phenol agent found in the skin of grapes, peanuts and pine bark [57]. It has been reported to have antioxidant, anti-inflammatory and anticancer effects [65]. Observational population studies in humans found that high ingestion of resveratrol was inversely associated with cancers such as breast and oesophageal cancer.

However, most studies in the literature on the use of resveratrol in cancer have been carried out in mouse models. It has been used in combination with other supplements such as curcumin and quercetin, and these in vivo studies conclude that resveratrol supplementation may promote tumour cell apoptosis and also attenuate proteolysis, thus ameliorating some effects of cancer cachexia [66,67].

### 2.11. Capsaicin and Boswellia Serrata

Similarly, other natural products known to have anti-inflammatory properties are capsaicin and Boswellia serrata. Little is known about their potential benefits for use in cancer cachexia; however, they have been used alongside chemotherapy agents.

Capsaicin is the main active ingredient found in chili peppers [31]. It has been tested alongside the widely used chemotherapeutic agent cisplatin to alleviate chemotherapy-induced muscle loss and atrophy. There may be some utility in capsaicin in alleviating chemotherapy-induced muscle loss, but further studies are needed.

Acetyl-keto-boswellic acid is extracted from the frankincence of the Boswellia species tree. It has been shown to have potential anticancer properties [68]. In in vitro studies and animal models, boswellic acids have been shown to inhibit pro-inflammatory enzyme synthesis [69]. In an in vitro study by Al-Bahlani et al., boswellic acid was shown to enhance gastric cancer cells to cisplatin-induced apoptosis. Sun et al. demonstrated that boswellic acid is able to promote apoptosis of cancer cells in vitro [68,70]. No direct use in cancer cachexia has been utilised in the literature, but given its anti-inflammatory properties, this plant extract should be further researched.

## 3. Conclusions

Cancer cachexia presents a significant challenge in the management of patients with gastric cancer, impacting both quality of life and treatment outcomes. Current therapeutic approaches focus on reducing systemic inflammation and preventing muscle loss through nutritional interventions, appetite stimulation and anti-inflammatory medications. However, the efficacy of pharmacological treatments is limited by adverse effects and potential interactions with chemotherapy agents. In this context, natural anti-inflammatory products emerge as promising adjunctive therapies for gastric cancer cachexia.

## 4. Future Directions

Future research in gastric cancer cachexia will aim to further understand the mechanisms underlying the metabolic and inflammatory pathophysiology of the disease. Current treatment strategies are limited, and the establishment of new treatments is dependent on the development of rigorous clinical trials that are expensive and time-consuming. The role of natural anti-inflammatory products as adjunctive therapies has the potential to improve patient outcomes with encouraging preclinical evidence; however, clinical evidence is scant.

Fatty acids, particularly omega-3 fatty acids derived from fish oil, have shown potential in reducing inflammation and preserving muscle mass in patients with gastric cancer. Further research must aim to standardise dosing and treatment duration. Probiotics, by modulating gut microbiota and reducing systemic inflammation, offer a novel approach to mitigating cachexia-related complications; however, clinical trials are needed to validate efficacy and safety in patients with gastric cancer cachexia. Amino acids have been demonstrated to decrease muscle wasting in certain cohorts; however, their efficacy in gastric cancer cachexia patients should be demonstrated in rigorous clinical trials.

Curcumin, fucoidan, epigallocatechin-3-gallate (EGCG) and ginger represent additional natural compounds with anti-inflammatory and anti-cachectic properties. While preclinical studies have shown promising results, clinical trials are needed to validate their efficacy and safety in gastric cancer cachexia. There are several challenges to be overcome such as variable bioavailabilities, potential interactions with other medications and supplements and ascertaining optimal delivery routes. Future research should aim to further understand the mechanisms of action of these compounds in the pre-clinical setting, as well as demonstrate safety and efficacy through well-designed clinical trials.

The multifactorial nature of gastric cancer cachexia necessitates a comprehensive approach integrating conventional therapies with novel strategies targeting systemic inflammation and muscle preservation. Natural anti-inflammatory products offer a promising avenue for improving outcomes in patients with gastric cancer by addressing the complex interplay of metabolic and inflammatory processes underlying cachexia.

## Figures and Tables

**Table 1:** Naturally occurring anti-inflammatory compounds; their dietary sources and proposed mechanisms of action. ↓ and ↑ respectively indicate reduction and increase in levels.

Compound	Major Dietary Sources	Proposed Mechanisms
Essential fatty acids	Nuts, seeds, fish oil	↓pro-inflammatory cytokine production ↑chemotherapy tolerance
Probiotics	Fermented foods including kimchi, kombucha, sauerkraut, kefir	↓gut dysbiosis ↑chemotherapy tolerance
Essential amino acids	Meat, fish, eggs, nuts, seeds	↑muscle anabolism/↓muscle catabolism
Curcumin	Turmeric (*Curcuma longa*) Other turmeric species	↓pro-inflammatory cytokine production ↑appetite
Fucoidan	Seaweed (kombu, wakame) Seafood (abalone, sea urchin)	↑chemotherapy tolerance
Epigallocatechin-3-gallate (EGCG)	Green tea White tea	Unknown
Ginger	Ginger (*Zingiber officinale Roscoe)*	↓nausea

## References

[1] Guan W.-L., He Y., Xu R.-H. (2023). Gastric Cancer Treatment: Recent Progress and Future Perspectives. J. Hematol. Oncol..

[2] Conti C.B., Agnesi S., Scaravaglio M., Masseria P., Dinelli M.E., Oldani M., Uggeri F. (2023). Early Gastric Cancer: Update on Prevention, Diagnosis and Treatment. Int. J. Environ. Res. Public Health.

[3] Mocan L. (2021). Surgical Management of Gastric Cancer: A Systematic Review. J. Clin. Med..

[4] Stomach Cancer Survival Rates and Statistics—NCI. https://www.cancer.gov/types/stomach/survival.

[5] Mizukami T., Piao Y. (2021). Role of Nutritional Care and General Guidance for Patients with Advanced or Metastatic Gastric Cancer. Future Oncol..

[6] Baracos V.E., Martin L., Korc M., Guttridge D.C., Fearon K.C.H. (2018). Cancer-Associated Cachexia. Nat. Rev. Dis. Primers.

[7] Brown L.R., Laird B.J.A., Wigmore S.J., Skipworth R.J.E. (2022). Understanding Cancer Cachexia and Its Implications in Upper Gastrointestinal Cancers. Curr. Treat. Options Oncol..

[8] Ni J., Zhang L. (2020). Cancer Cachexia: Definition, Staging, and Emerging Treatments. Cancer Manag. Res..

[9] Definition and Classification of Cancer Cachexia: An International Consensus—The Lancet Oncology. https://www.thelancet.com/journals/lanonc/article/PIIS1470-2045(10)70218-7/abstract.

[10] Fearon K., Strasser F., Anker S.D., Bosaeus I., Bruera E., Fainsinger R.L., Jatoi A., Loprinzi C., MacDonald N., Mantovani G. (2011). Definition and Classification of Cancer Cachexia: An International Consensus. Lancet Oncol..

[11] Braha A., Albai A., Timar B., Negru Ș., Sorin S., Roman D., Popovici D. (2022). Nutritional Interventions to Improve Cachexia Outcomes in Cancer-A Systematic Review. Medicina.

[12] Isenring E.A., Teleni L. (2013). Nutritional Counseling and Nutritional Supplements: A Cornerstone of Multidisciplinary Cancer Care for Cachectic Patients. Curr. Opin. Support. Palliat. Care.

[13] Panebianco C., Villani A., Potenza A., Favaro E., Finocchiaro C., Perri F., Pazienza V. (2023). Targeting Gut Microbiota in Cancer Cachexia: Towards New Treatment Options. Int. J. Mol. Sci..

[14] Grundmann O., Yoon S.L., Williams J.J. (2020). Editor’s Pick: Cachexia in Patients with Gastrointestinal Cancers: Contributing Factors, Prevention, and Current Management Approaches. EMJ Gastroenterol..

[15] Dijksterhuis W.P.M., Latenstein A.E.J., van Kleef J.J., Verhoeven R.H.A., de Vries J.H.M., Slingerland M., Steenhagen E., Heisterkamp J., Timmermans L.M., de van der Schueren M.A.E. (2021). Cachexia and Dietetic Interventions in Patients With Esophagogastric Cancer: A Multicenter Cohort Study. J. Natl. Compr. Cancer Netw..

[16] Roeland E.J., Bohlke K., Baracos V.E., Bruera E., Del Fabbro E., Dixon S., Fallon M., Herrstedt J., Lau H., Platek M. (2020). Management of Cancer Cachexia: ASCO Guideline. J. Clin. Oncol..

[17] Herremans K.M., Riner A.N., Cameron M.E., Trevino J.G. (2019). The Microbiota and Cancer Cachexia. Int. J. Mol. Sci..

[18] Wallace J.M. (2002). Nutritional and Botanical Modulation of the Inflammatory Cascade—Eicosanoids, Cyclooxygenases, and Lipoxygenases—As an Adjunct in Cancer Therapy. Integr. Cancer Ther..

[19] Office of Dietary Supplements—Dietary Supplements: What You Need to Know. https://ods.od.nih.gov/factsheets/WYNTK-Consumer/.

[20] Han Y., Kim H.I., Park J. (2023). The Role of Natural Products in the Improvement of Cancer-Associated Cachexia. Int. J. Mol. Sci..

[21] Simopoulos A.P. (2002). Omega-3 Fatty Acids in Inflammation and Autoimmune Diseases. J. Am. Coll. Nutr..

[22] Lomax A., Calder P. (2009). Probiotics, Immune Function, Infection and Inflammation: A Review of the Evidence from Studies Conducted in Humans. Curr. Pharm. Des..

[23] Hill C., Guarner F., Reid G., Gibson G.R., Merenstein D.J., Pot B., Morelli L., Canani R.B., Flint H.J., Salminen S. (2014). Expert Consensus Document. The International Scientific Association for Probiotics and Prebiotics Consensus Statement on the Scope and Appropriate Use of the Term Probiotic. Nat. Rev. Gastroenterol. Hepatol..

[24] Carbone J.W., Pasiakos S.M. (2019). Dietary Protein and Muscle Mass: Translating Science to Application and Health Benefit. Nutrients.

[25] Tipton K.D. (2011). Efficacy and Consequences of Very-High-Protein Diets for Athletes and Exercisers. Proc. Nutr. Soc..

[26] Oelkrug C., Lange C.M., Wenzel E., Fricke S., Hartke M., Simasi J., Schubert A. (2014). Analysis of the Tumoricidal and Anti-Cachectic Potential of Curcumin. Anticancer Res..

[27] Wu C.-J., Yeh T.-P., Wang Y.-J., Hu H.-F., Tsay S.-L., Liu L.-C. (2022). Effectiveness of Fucoidan on Supplemental Therapy in Cancer Patients: A Systematic Review. Healthcare.

[28] Shirai Y., Okugawa Y., Hishida A., Ogawa A., Okamoto K., Shintani M., Morimoto Y., Nishikawa R., Yokoe T., Tanaka K. (2017). Fish Oil-Enriched Nutrition Combined with Systemic Chemotherapy for Gastrointestinal Cancer Patients with Cancer Cachexia. Sci. Rep..

[29] Wang H., Lai Y.-J., Chan Y.-L., Li T.-L., Wu C.-J. (2011). Epigallocatechin-3-Gallate Effectively Attenuates Skeletal Muscle Atrophy Caused by Cancer Cachexia. Cancer Lett..

[30] Bhargava R., Chasen M., Elten M., MacDonald N. (2020). The Effect of Ginger (Zingiber Officinale Roscoe) in Patients with Advanced Cancer. Support. Care Cancer.

[31] Huang K., Chiang Y., Huang T., Chen H., Lin P., Ali M., Hsia S. (2023). Capsaicin Alleviates Cisplatin-induced Muscle Loss and Atrophy in Vitro and in Vivo. J. Cachexia Sarcopenia Muscle.

[32] Yoon S.L., Grundmann O. (2023). Relevance of Dietary Supplement Use in Gastrointestinal-Cancer-Associated Cachexia. Nutrients.

[33] Coniglio S., Shumskaya M., Vassiliou E. (2023). Unsaturated Fatty Acids and Their Immunomodulatory Properties. Biology.

[34] Calder P.C. (2017). Omega-3 Fatty Acids and Inflammatory Processes: From Molecules to Man. Biochem. Soc. Trans..

[35] Persson C., Glimelius B., Rönnelid J., Nygren P. (2005). Impact of Fish Oil and Melatonin on Cachexia in Patients with Advanced Gastrointestinal Cancer: A Randomized Pilot Study. Nutrition.

[36] Aquila G., Re Cecconi A.D., Brault J.J., Corli O., Piccirillo R. (2020). Nutraceuticals and Exercise against Muscle Wasting during Cancer Cachexia. Cells.

[37] Burns C.P., Halabi S., Clamon G., Kaplan E., Hohl R.J., Atkins J.N., Schwartz M.A., Wagner B.A., Paskett E. (2004). Phase II Study of High-Dose Fish Oil Capsules for Patients with Cancer-Related Cachexia. Cancer.

[38] O’Hara A.M., Shanahan F. (2006). The Gut Flora as a Forgotten Organ. EMBO Rep..

[39] Ríos-Covián D., Ruas-Madiedo P., Margolles A., Gueimonde M., De Los Reyes-Gavilán C.G., Salazar N. (2016). Intestinal Short Chain Fatty Acids and Their Link with Diet and Human Health. Front. Microbiol..

[40] Jiang Y., Guo C., Zhang D., Zhang J., Wang X., Geng C. (2014). The Altered Tight Junctions: An Important Gateway of Bacterial Translocation in Cachexia Patients with Advanced Gastric Cancer. J. Interferon Cytokine Res..

[41] Huang F., Li S., Chen W., Han Y., Yao Y., Yang L., Li Q., Xiao Q., Wei J., Liu Z. (2023). Postoperative Probiotics Administration Attenuates Gastrointestinal Complications and Gut Microbiota Dysbiosis Caused by Chemotherapy in Colorectal Cancer Patients. Nutrients.

[42] Zaharuddin L., Mokhtar N.M., Muhammad Nawawi K.N., Raja Ali R.A. (2019). A Randomized Double-Blind Placebo-Controlled Trial of Probiotics in Post-Surgical Colorectal Cancer. BMC Gastroenterol..

[43] Varian B.J., Goureshetti S., Poutahidis T., Lakritz J.R., Levkovich T., Kwok C., Teliousis K., Ibrahim Y.M., Mirabal S., Erdman S.E. (2016). Beneficial Bacteria Inhibit Cachexia. Oncotarget.

[44] Bindels L.B., Neyrinck A.M., Claus S.P., Le Roy C.I., Grangette C., Pot B., Martinez I., Walter J., Cani P.D., Delzenne N.M. (2016). Synbiotic Approach Restores Intestinal Homeostasis and Prolongs Survival in Leukaemic Mice with Cachexia. ISME J..

[45] Redman M.G., Ward E.J., Phillips R.S. (2014). The Efficacy and Safety of Probiotics in People with Cancer: A Systematic Review. Ann. Oncol..

[46] Deutz N.E.P., Safar A., Schutzler S., Memelink R., Ferrando A., Spencer H., Van Helvoort A., Wolfe R.R. (2011). Muscle Protein Synthesis in Cancer Patients Can Be Stimulated with a Specially Formulated Medical Food. Clin. Nutr..

[47] Johal J., Han C.Y., Joseph R., Munn Z., Agbejule O.A., Crawford-Williams F., Wallen M.P., Chan R.J., Hart N.H. (2022). Dietary Supplements in People with Metastatic Cancer Who Are Experiencing Malnutrition, Cachexia, Sarcopenia, and Frailty: A Scoping Review. Nutrients.

[48] Cencioni C., Trestini I., Piro G., Bria E., Tortora G., Carbone C., Spallotta F. (2022). Gastrointestinal Cancer Patient Nutritional Management: From Specific Needs to Novel Epigenetic Dietary Approaches. Nutrients.

[49] Cangiano C., Laviano A., Meguid M.M., Mulieri M., Conversano L., Preziosa I., Rossi-Fanelli F. (1996). Effects of Administration of Oral Branched-Chain Amino Acids on Anorexia and Caloric Intake in Cancer Patients. J. Natl. Cancer Inst..

[50] Does Branched-Chain Amino Acids Supplementation Modulate Skeletal Muscle Remodeling through Inflammation Modulation? Possible Mechanisms of Action. https://www.hindawi.com/journals/jnme/2012/136937/.

[51] Tayek J.A., Bistrian B.R., Hehir D.J., Martin R., Moldawer L.L., Blackburn G.L. (1986). Improved Protein Kinetics and Albumin Synthesis by Branched Chain Amino Acid-Enriched Total Parenteral Nutrition in Cancer Cachexia. A Prospective Randomized Crossover Trial. Cancer.

[52] Ragni M., Fornelli C., Nisoli E., Penna F. (2022). Amino Acids in Cancer and Cachexia: An Integrated View. Cancers.

[53] Chaiworramukkul A., Seetalarom K., Saichaemchan S., Prasongsook N. (2022). A Double-Blind, Placebo-Controlled Randomized Phase IIa Study: Evaluating the Effect of Curcumin for Treatment of Cancer Anorexia–Cachexia Syndrome in Solid Cancer Patients. Asian Pac. J. Cancer Prev..

[54] Stohs S.J., Chen O., Ray S.D., Ji J., Bucci L.R., Preuss H.G. (2020). Highly Bioavailable Forms of Curcumin and Promising Avenues for Curcumin-Based Research and Application: A Review. Molecules.

[55] Kanai M., Otsuka Y., Otsuka K., Sato M., Nishimura T., Mori Y., Kawaguchi M., Hatano E., Kodama Y., Matsumoto S. (2013). A Phase I Study Investigating the Safety and Pharmacokinetics of Highly Bioavailable Curcumin (Theracurmin) in Cancer Patients. Cancer Chemother. Pharmacol..

[56] Cheng A.L., Hsu C.H., Lin J.K., Hsu M.M., Ho Y.F., Shen T.S., Ko J.Y., Lin J.T., Lin B.R., Ming-Shiang W. (2001). Phase I Clinical Trial of Curcumin, a Chemopreventive Agent, in Patients with High-Risk or Pre-Malignant Lesions. Anticancer Res..

[57] Li W., Swiderski K., Murphy K.T., Lynch G.S. (2022). Role for Plant-Derived Antioxidants in Attenuating Cancer Cachexia. Antioxidants.

[58] Anisha G.S., Padmakumari S., Patel A.K., Pandey A., Singhania R.R. (2022). Fucoidan from Marine Macroalgae: Biological Actions and Applications in Regenerative Medicine, Drug Delivery Systems and Food Industry. Bioengineering.

[59] Wang Y., Xing M., Cao Q., Ji A., Liang H., Song S. (2019). Biological Activities of Fucoidan and the Factors Mediating Its Therapeutic Effects: A Review of Recent Studies. Mar. Drugs.

[60] Ikeguchi M., Saito H., Miki Y., Kimura T. (2015). Effect of Fucoidan Dietary Supplement on the Chemotherapy Treatment of Patients with Unresectable Advanced Gastric Cancer. J. Cancer Ther..

[61] Furniturewalla A., Barve K. (2022). Approaches to Overcome Bioavailability Inconsistencies of Epigallocatechin Gallate, a Powerful Anti-Oxidant in Green Tea. Food Chem. Adv..

[62] Lete I., Alluέ J. (2016). The Effectiveness of Ginger in the Prevention of Nausea and Vomiting during Pregnancy and Chemotherapy. Integr. Med. Insights.

[63] Velázquez K.T., Enos R.T., Narsale A.A., Puppa M.J., Davis J.M., Murphy E.A., Carson J.A. (2014). Quercetin Supplementation Attenuates the Progression of Cancer Cachexia in Apc Mice. J. Nutr..

[64] Miles S.L., McFarland M., Niles R.M. (2014). Molecular and Physiological Actions of Quercetin: Need for Clinical Trials to Assess Its Benefits in Human Disease. Nutr. Rev..

[65] Meng X., Zhou J., Zhao C.-N., Gan R.-Y., Li H.-B. (2020). Health Benefits and Molecular Mechanisms of Resveratrol: A Narrative Review. Foods.

[66] Penedo-Vázquez A., Duran X., Mateu J., López-Postigo A., Barreiro E. (2021). Curcumin and Resveratrol Improve Muscle Function and Structure through Attenuation of Proteolytic Markers in Experimental Cancer-Induced Cachexia. Molecules.

[67] Li C., Xu Y., Zhang J., Zhang Y., He W., Ju J., Wu Y., Wang Y. (2023). The Effect of Resveratrol, Curcumin and Quercetin Combination on Immuno-Suppression of Tumor Microenvironment for Breast Tumor-Bearing Mice. Sci. Rep..

[68] Al-Bahlani S., Burney I.A., Al-Dhahli B., Al-Kharusi S., Al-Kharousi F., Al-Kalbani A., Ahmed I. (2020). Boswellic Acid Sensitizes Gastric Cancer Cells to Cisplatin-Induced Apoptosis via P53-Mediated Pathway. BMC Pharmacol. Toxicol..

[69] Di Lorenzo C., Dell’agli M., Badea M., Dima L., Colombo E., Sangiovanni E., Restani P., Bosisio E. (2013). Plant Food Supplements with Anti-Inflammatory Properties: A Systematic Review (II). Crit. Rev. Food Sci. Nutr..

[70] Sun M.-X., He X.-P., Huang P.-Y., Qi Q., Sun W.-H., Liu G.-S., Hua J. (2020). Acetyl-11-Keto-β-Boswellic Acid Inhibits Proliferation and Induces Apoptosis of Gastric Cancer Cells through the Phosphatase and Tensin Homolog /Akt/ Cyclooxygenase-2 Signaling Pathway. World J. Gastroenterol..

